# Rutin Inhibits Ox-LDL-Mediated Macrophage Inflammation and Foam Cell Formation by Inducing Autophagy and Modulating PI3K/ATK Signaling

**DOI:** 10.3390/molecules27134201

**Published:** 2022-06-29

**Authors:** Ben Li, Yumeng Ji, Chenlong Yi, Xufeng Wang, Chaoyang Liu, Chufan Wang, Xiaohu Lu, Xiaohan Xu, Xiaowei Wang

**Affiliations:** 1 Department of Cardiovascular Surgery, The First Affiliated Hospital of Nanjing Medical University, Nanjing 210000, China; liben1966@163.com (B.L.); jiyumeng@stu.njmu.edu.cn (Y.J.); wxf37409878@163.com (X.W.); liuzhy_21@stu.njmu.edu.cn (C.L.); 13951650148@163.com (C.W.); lxhtiger1980@163.com (X.L.); 2 The First Clinical School of Medicine, Nanjing Medical University, Nanjing 210000, China; 3 Department of Cardiovascular Surgery, Yangzhou First People’s Hospital, Yangzhou 225000, China; yichenlong_001@163.com

**Keywords:** rutin, atherosclerosis, RAW264.7, inflammation, autophagy

## Abstract

Atherosclerosis (AS) is one of the leading causes of death among the elderly, and is primarily caused by foam cell generation and macrophage inflammation. Rutin is an anti-inflammatory, anti-oxidant, anti-allergic, and antiviral flavonoid molecule, known to have anti-atherosclerotic and autophagy-inducing properties, but its biological mechanism remains poorly understood. In this study, we uncovered that rutin could suppress the generation of inflammatory factors and reactive oxygen species (ROS) in ox-LDL-induced M2 macrophages and enhance their polarization. Moreover, rutin could decrease foam cell production, as shown by oil red O staining. In addition, rutin could increase the number of autophagosomes and the LC3II/I ratio, while lowering p62 expression. Furthermore, rutin could significantly inhibit the PI3K/ATK signaling pathway. In summary, rutin inhibits ox-LDL-mediated macrophage inflammation and foam cell formation by inducing autophagy and modulating PI3K/ATK signaling, showing potential in treating atherosclerosis.

## 1. Introduction

The most common cause of coronary heart disease, stroke, and peripheral vascular disease is atherosclerosis (AS). It is also one of the leading causes of death among the elderly [1]. Atherosclerotic lesions are characterized by the accumulation of lipids and other blood components in the artery intima, the proliferation and expansion of smooth muscle cells and collagen fibers, the formation of atherosclerotic fat-containing necrotic lesions, and vascular wall sclerosis. Inflammation has been implicated in the initiation and progression of atherosclerotic plaques in numerous studies [2]. Macrophages have the ability to ingest oxidized low-density lipoprotein cholesterol, forming foam cells as a result. Foam cells are common pathogenic cells found in atherosclerotic plaques, and their development exacerbates the severity of atherosclerotic lesions [3]. As a result, inhibiting foam cell generation and macrophage inflammation in atherosclerotic lesions may provide an attractive therapeutic and preventive strategy for managing atherosclerosis.

Autophagy is the process by which a cell swallows its cytoplasmic proteins or organelles, encapsulating them in vesicles followed by fusion with lysosomes to form autophagic lysosomes to digest its contents, thereby meeting the cell′s metabolic requirements and renewing specific organelles [4]. Autophagy plays a crucial role in atherosclerosis. Autophagy can be facilitated by inhibiting the PI3K/Akt signaling pathway, which, in turn, decreases foam cell production [5]. Inhibition of the PI3K/Akt/mTOR axis can enhance macrophage autophagy, thereby playing a protective role in regulating lipid accumulation and polarization transformation [6]. Furthermore, mTOR is implicated in the formation of atherosclerotic plaques. While mTOR promotes foam cell production, rapamycin, an mTOR inhibitor, can suppress mTOR activity in macrophages while also decreasing plaque inflammation [5]. Furthermore, inhibiting mTOR can help to stabilize atherosclerotic plaques by increasing autophagy [7]. Inhibiting the PI3K/Akt/mTOR pathway increases foam cell cholesterol export and promotes autophagy, both of which help to diminish plaque inflammation [6]. We, thus, believe that macrophage autophagy could be a future direction in preventing and treating atherosclerosis.

Rutin is an anti-inflammatory, anti-oxidant, anti-allergic, and antiviral flavonoid molecule [8]. Moreover, rutin is known to have anti-atherosclerotic and autophagy-inducing properties [9,10]. Although rutin has been shown to suppress the production of foam cells, its biological mechanism remains poorly understood.

The phenotypic differentiation of macrophages significantly influences the atherosclerotic process. Differentiation of macrophages to the M1 phenotype plays a pro-inflammatory role in the progression of atherosclerosis, whereas the differentiation of macrophages to the M2 phenotype plays an anti-inflammatory role in the progression of atherosclerosis. In this study, we chose the most widely used iNOS as the M1 macrophage marker and Arg1 as the M2 macrophage marker. We explored whether rutin could inhibit the inflammatory response by inhibiting the polarization of macrophages to the M1 type and promoting their polarization to the M2 type by detecting the protein levels of both.

This study aimed to determine whether rutin could inhibit ox-LDL-mediated macrophage inflammation and foam cell production by activating autophagy and modulating the PI3K/ATK signaling pathway.

## 2. Results

### 2.1. Rutin Can Improve the Viability of RAW264.7 Cells

**Figure 1:**
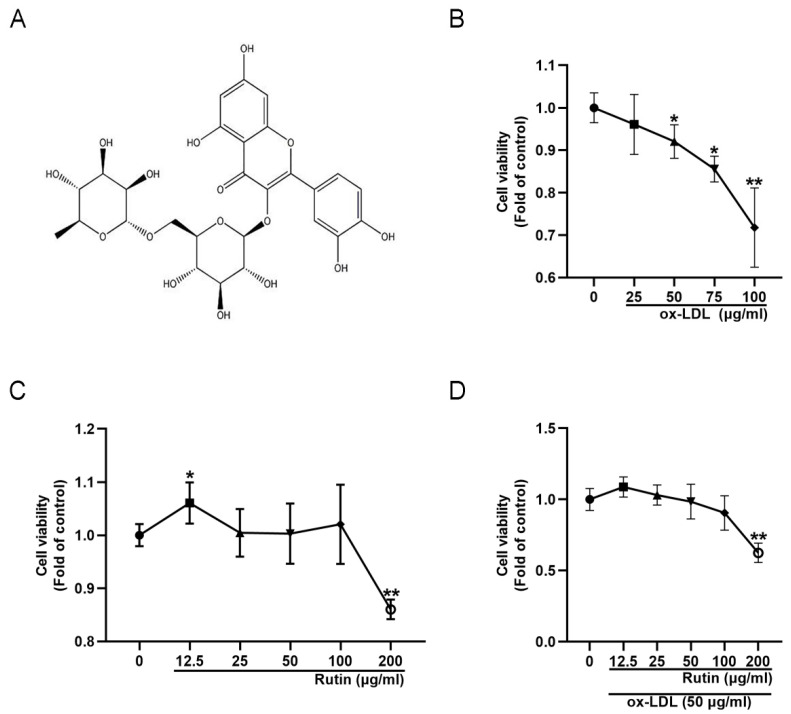
Rutin can help RAW264.7 cells survive after being exposed to ox-LDL. (**A**) Rutin′s chemical formula. (**B**) Viability of RAW264.7 cells treated with ox-LDL. (**C**) RAW264 cell viability after rutin treatment. (**D**) Viability of RAW264.7 cells treated with rutin and ox-LDL. * *p* < 0.05; ** *p* < 0.01. Data are provided as mean ± SD.

To generate a macrophage-derived foam cell model, varying concentrations of ox-LDL were used to induce RAW264.7 cells. According to the results of the CCK-8 experiment, 50 μg/mL ox-LDL could significantly reduce cell viability (Figure 1B). Thus, to induce the formation of foam cells in subsequent experiments, 50 μg/mL ox-LDL was utilized, which is also the dose employed in other previous reports [11,12]. Following that, the effect of rutin on the viability of RAW264.7 cells was determined. At 12.5 μg/mL rutin, cell viability increased significantly, but drastically decreased at a concentration of 200 μg/mL (Figure 1C). To assess rutin′s protective effect on ox-LDL-induced foam cells, RAW264.7 cells were pretreated with different concentrations of rutin for 24 h prior to being stimulated with 50 μg/mL ox-LDL. The results showed that unlike the high concentration of 200 μg/mL rutin, the low concentration of rutin (12.5, 25, 50, 100 μg/mL) could reduce the cell viability damage caused by 50 μg/mL ox-LDL in RAW264.7 cells (Figure 1D). Therefore, the optimal dose of rutin used in subsequent experiments was 12.5 μg/mL. Taken together, these data suggested that rutin could improve the viability of RAW264.7 cells.

### 2.2. Rutin Can Inhibit Macrophage Inflammation

**Figure 2:**
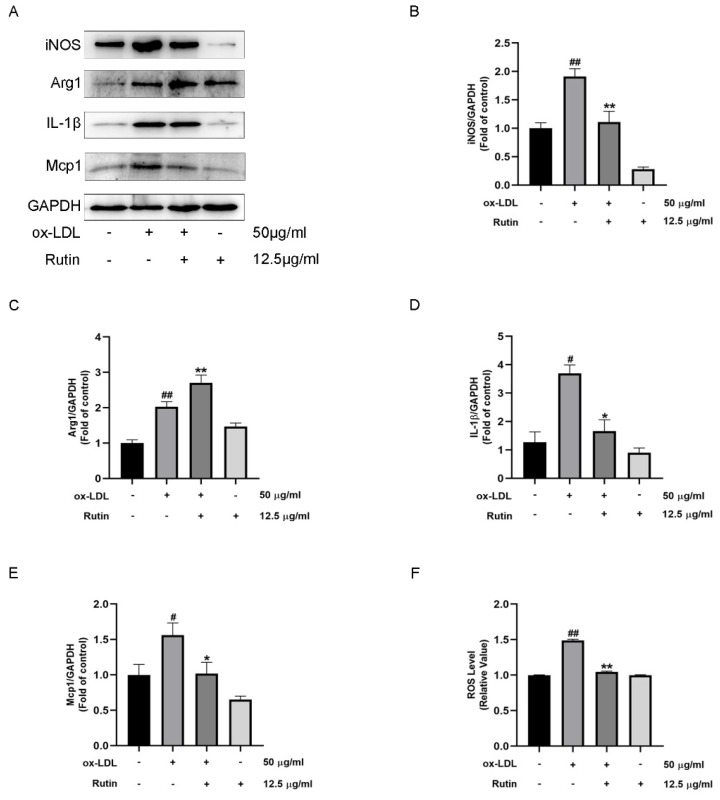
Rutin has the ability to reduce macrophage inflammation. RAW264.7 cells were stimulated for 24 h with 12.5 μg/mL rutin with or without 50 μg/mL ox-LDL therapy. (**A**–**E**) Western Blot was used to detect the expression levels of iNOS, ARG1, IL-1β and Mcp1. (**F**) DCFH-DA staining was used to quantify intracellular ROS generation. # *p* < 0.05, ## *p* < 0.01 vs. control group; * *p* < 0.05, ** *p* < 0.01 vs. ox-LDL-treated group. All data are shown as the mean ± SD of three independent trials, each of which was carried out in triplicate.

To determine rutin′s anti-inflammatory properties, the expression levels of M1 and M2 macrophage markers were investigated. Results showed that Rutin treatment could significantly boost Arg1 expression, while suppressing iNOS, IL-1β, and Mcp1 expression levels (Figure 2A–E). Furthermore, reactive oxygen species (ROS) generation was significantly decreased in vitro (Figure 2F). These results suggested that rutin could inhibit macrophage inflammation.

### 2.3. Rutin Can Promote Autophagy in Macrophages

**Figure 3:**
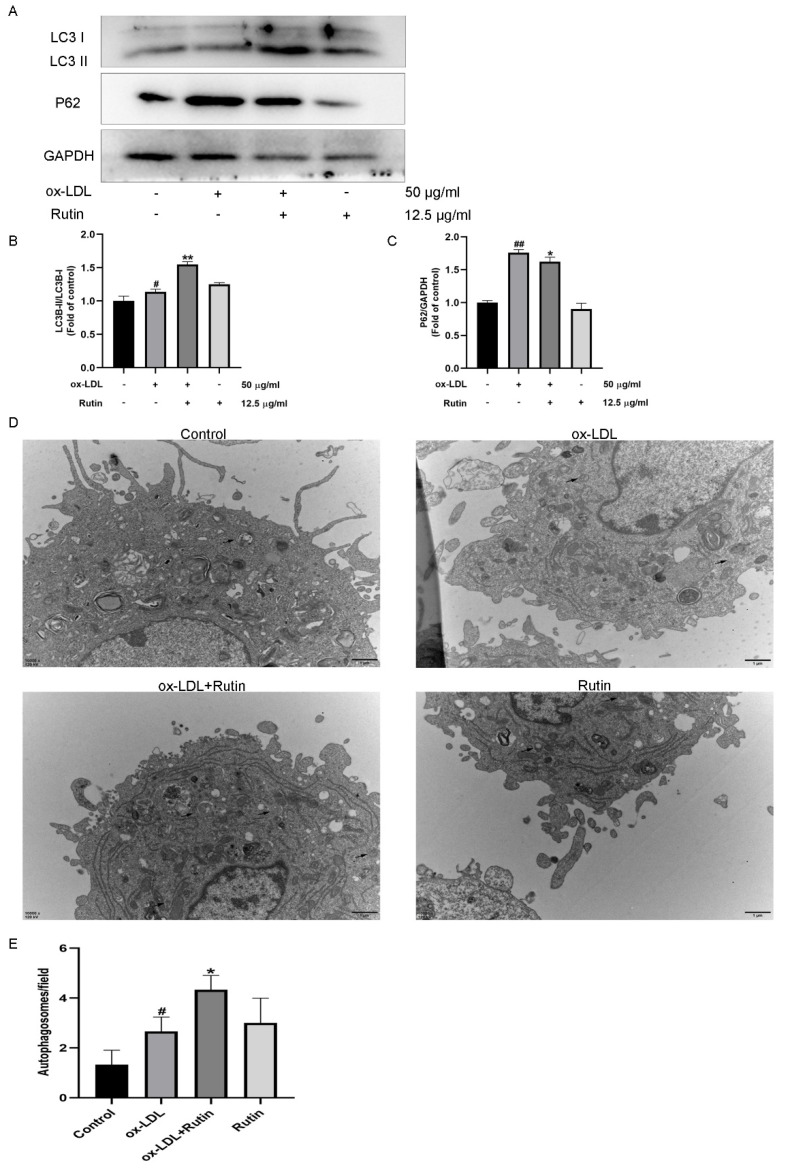
Rutin can promote autophagy in macrophages. RAW264.7 cells were stimulated for 24 h with 12.5 μg/mL rutin with or without 50 μg/mL ox-LDL therapy. (**A**–**C**) Western blot was used to detect the levels of LC3II/LC3I, P62 protein expression. (**D**,**E**) Representative TEM images and quantification of autophagosomes (arrows) in each group. # *p* < 0.05, ## *p* < 0.01 vs. control group; * *p* < 0.05, ** *p* < 0.01 vs. ox-LDL-treated group. The data are presented as mean ± SD.

In previous studies, autophagy was shown to play a substantial role in the pathogenesis of atherosclerosis [13,14]. To that end, we investigated the possible association between rutin and autophagy-related proteins using western blot analysis and electron microscopy. According to the findings, rutin increased the LC3II/LC3I ratio but decreased the protein expression of P62 (Figure 3A–C). Moreover, rutin could also boost the number of autophagosomes in macrophages (Figure 3D,E). These data suggested that rutin could promote autophagy in macrophages.

### 2.4. Rutin Can Inhibit the Production of Foam Cells Induced by ox-LDL

**Figure 4:**
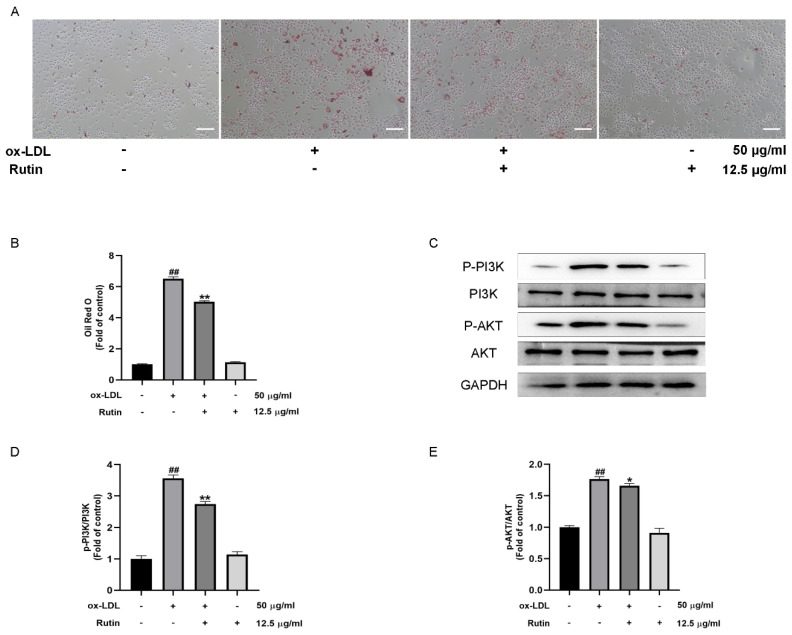
Rutin suppresses the PI3K/AKT signaling pathway and ox-LDL-induced foam cell formation. RAW264.7 cells were stimulated for 24 h with 12.5 μg/mL rutin with or without 50 μg/mL ox-LDL therapy. (**A**,**B**) Oil red O staining (Scale bars = 100 µm). (**C**–**E**) P-PI3K, PI3K, P-AKT, AKT expression. ## *p* < 0.01 vs. control group; * *p* < 0.05, ** *p* < 0.01 vs. ox-LDL-treated group. Data are presented as mean ± SD.

Atherosclerosis is associated with elevated oxygenated low-density lipoprotein levels (ox-LDL). It has been shown to stimulate the formation of atherosclerotic foam cells and contribute to the onset and progression of atherosclerosis [15,16]. Oil Red O is a lipid-soluble dye that stains neutral triglycerides and lipids and is used to visualize lipid content in macrophages, as well as in foam cells. The oil red O stain findings revealed that ox-LDL treatment could enhance the accumulation of lipids, while rutin treatment inhibited the formation of foam cells induced by ox-LDL (Figure 4A,B). Therefore, these findings suggested that rutin could inhibit the production of foam cells induced by ox-LDL.

### 2.5. Rutin Can Inhibit the PI3K/AKT Signaling Pathway

Numerous studies have associated the PI3K/AKT signaling pathway with inflammation and autophagy [17,18]. However, it is unknown whether rutin can attenuate ox-LDL-mediated macrophage inflammation and foam cell formation by modulating the PI3K/ATK signaling. Our results indicated that the expression of phosphorylated-activated PI3K/AKT was significantly higher in ox-LDL-induced macrophages in comparison with the control group. Furthermore, the addition of rutin could inhibit the activation of the PI3K/AKT pathway in ox-LDL-induced macrophages (Figure 4C–E). The above findings revealed that rutin could substantially inhibit the PI3K/AKT signaling pathway. Taken together, these results suggested that rutin could inhibit the PI3K/AKT signaling pathway. 

### 2.6. Rutin Can Reduce Macrophage Inflammation and Foam Cell Production via Modulating PI3K/ATK Signaling and Activating Autophagy

**Figure 5:**
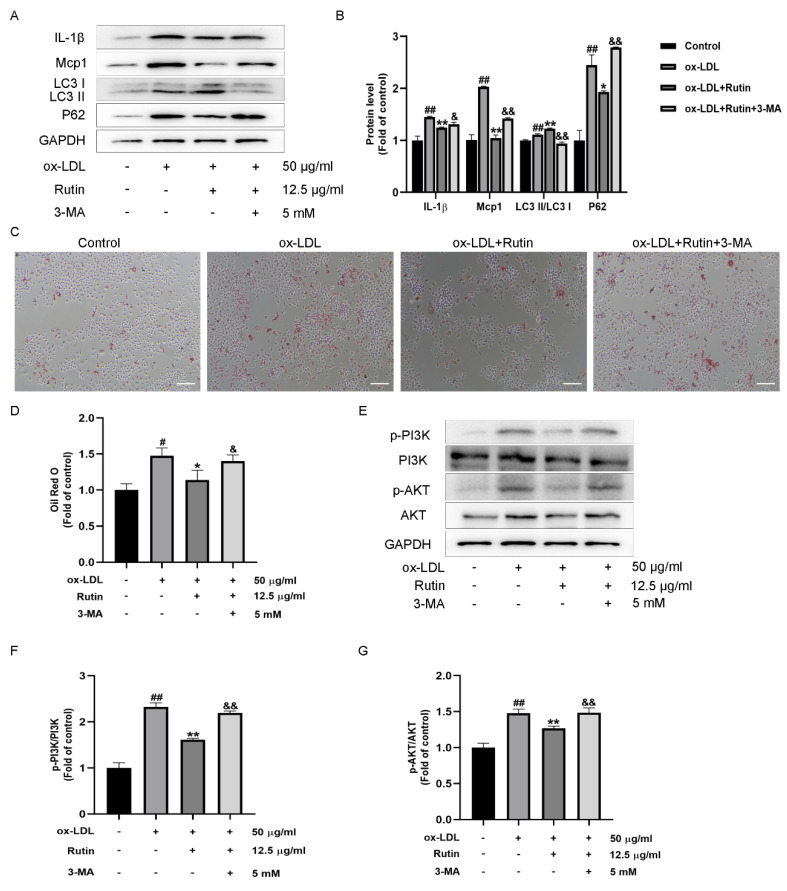
Rutin inhibits macrophage inflammation and foam cell formation via activating autophagy and regulating PI3K/ATK signaling. RAW264.7 cells were stimulated for 24 h with 12.5 μg/mL rutin and subsequently treated for 24 h with 50 μg/mL ox-LDL in the presence or absence of the autophagy inhibitor 3-MA. (**A**,**B**) Levels of IL-1β, Mcp1, LC3II/LC3I and P62 expression. (**C**,**D**) Staining with oil red O (Scale bars = 100 µm). (**E**–**G**) P-PI3K, PI3K, P-AKT and AKT expression. # *p* < 0.05, ## *p* < 0.01 vs. control group; * *p* < 0.05, ** *p* < 0.01 vs. ox-LDL-treated group; & *p* < 0.05, && *p* < 0.01 vs. ox-LDL and rutin treatment group. Data are presented as mean ± SD.

The PI3K/AKT signaling pathway is essential for autophagy. Inhibiting the PI3K/AKT signaling pathway promotes autophagosome-lysosome fusion and degradation in RAW264.7 cells [19]. Moreover, inhibiting the PI3K/AKT signaling pathway induces autophagy, resulting in decreased macrophage inflammation and foam cell formation [5,20]. However, whether rutin inhibits PI3K/ATK signaling and reduces macrophage inflammation and foam cell production through triggering autophagy remains unclear. To demonstrate that autophagy is involved in rutin’s anti-inflammatory effects on macrophage inflammatory response and foam cell formation, the autophagy inhibitor 3-MA was utilized. The decreased LC3II/LC3I ratio and increased P62 expression indicated that 3-MA administration effectively suppressed autophagy compared with the rutin group. Additionally, after 3-MA administration, rutin’s anti-inflammatory activity was diminished, as shown by increased IL-1β and Mcp1 expression levels (Figure 5A,B). Importantly, pretreatment with 3-MA boosted foam cell production significantly (Figure 5C,D) and promoted PI3K/AKT signaling pathway activation (Figure 5E–G). Taken together, we showed that rutin suppressed ox-LDL-induced macrophage inflammation and foam cell formation via increasing autophagy and regulating the PI3K/ATK signaling pathway. Rutin could reduce macrophage inflammation and foam cell production via modulating PI3K/ATK signaling and activating autophagy.

## 3. Discussion

Even though statins and other anti-atherosclerotic medications are particularly effective at lowering LDL and can also suppress inflammation, regardless of their effect on lipids, new treatments are still needed to improve atherosclerosis prevention and therapy [21]. Rutin is a flavonoid found in a wide variety of plants. It possesses anti-inflammatory, anti-oxidant, neuroprotective, nephroprotective, and hepatoprotective properties [8]. Although studies have demonstrated that rutin has anti-atherosclerotic activity [10], the chemical mechanism by which it functions remains elusive. Our findings indicate that rutin has the potential to significantly reduce ox-LDL-induced macrophage inflammation and foam cell generation via autophagy activation and modulation of the PI3K/ATK signaling pathway, hinting that rutin may be used to treat atherosclerosis.

The formation of foam cells is a critical stage in the evolution of atherosclerosis [22]. Herein, we stimulated Raw264.7 cells with ox-LDL, resulting in decreased viability, and demonstrated that lipid accumulation could predict the successful development of the foam cell model using Oil Red O staining. Alternatively, rutin could inhibit ox-LDL-induced foam cell formation, validating rutin′s anti-atherosclerotic properties.

Inflammation affects both the occurrence and development of atherosclerotic plaques [2]. The phenotypic differentiation of macrophages has a significant influence on the atherosclerosis process. The differentiation of macrophages to the M1 phenotype has a proinflammatory effect in atherosclerosis advancement, while the differentiation of macrophages to the M2 phenotype has a role in atherosclerosis progression. Anti-inflammatory agents have been shown to reduce the progression of atherosclerosis [23]. In this study, we uncovered that rutin could inhibit the gene expression of IL-1β, Mcp1, iNOS, and other inflammatory mediators, while upregulating Arg1 expression. Together, this indicates that rutin could encourage macrophage differentiation to the M2 phenotype, thus suppressing inflammatory responses.

Inducing autophagy may be a therapeutic strategy for atherosclerosis, given its crucial role in atherosclerosis progression [7]. Autophagy can be facilitated by inhibiting the PI3K/Akt signaling pathway, which results in a decrease in the generation of foam cells [5]. Inhibition of the PI3K/Akt/mTOR axis enhances autophagy in macrophages, thereby exerting a protective role in regulating lipid accumulation and polarization transition [6]. According to our findings, rutin increased the LC3II/LC3I ratio but decreased the synthesis of P62 protein. Rutin could encourage macrophages to manufacture more autophagosomes. Furthermore, the autophagy inhibitor 3-MA could significantly increase lipid accumulation in RAW264.7 cells.

Autophagy is promoted by inhibiting the PI3K/AKT signaling pathway, which reduces macrophage inflammation and foam cell production [5,20]. Previous studies have shown that low-concentration rutin treatment can alleviate the cardiotoxic effect of pirarubicin on cardiomyocytes by activating the PI3K/AKT/mTOR signaling pathway [24]. In ox-LDL-stimulated macrophages, the addition of rutin reduced the activation of the PI3K/AKT pathway. However, the addition of 3-MA before rutin treatment dramatically boosted foam cell production and encouraged PI3K/AKT pathway activation.

## 4. Materials and Methods

### 4.1. Cells and Reagents

RAW264.7 mouse macrophages were obtained from the cell bank of the Chinese Academy of Sciences. Rutin was obtained from Shanghai Yuanye Biotechnology Co., Ltd., B20771, Shanghai, China, while ox-LDL was purchased from Guangzhou Yiyuan Biotechnology Co., Ltd., YB-002, Guangzhou, China. Glutaraldehyde at a concentration of 2.5% (Shanghai Yuanye Biotechnology Co., Ltd., R20510, Shanghai, China) was prepared. The primary antibodies against INOS, Arg1, IL-1β, and Mcp1 were obtained from Proteintech (Wuhan, China), while primary antibodies against P62, LC3, PI3K, p-PI3K, ATK, and p-ATK were bought from Cell Signaling Technology (Boston, MA, USA). Sigma-Aldrich provided the 3-methyladenine (3-MA) (St. Louis, MO, USA).

### 4.2. Cell Culture

RAW264.7 macrophages were cultivated at 37 °C in 5% CO_2_ in Dulbecco’s Modified Eagle’s Medium (DMEM) containing 10% fetal bovine serum (FBS). The cells employed in all experiments were passaged at least three times and no more than six times. Cells were separated into the following groups at random: Control, ox-LDL, ox-LDL + Rutin, Rutin, ox-LDL + Rutin + 3-MA.

### 4.3. Cell Counting Kit 8 Assays

The cells were seeded at a density of 2 × 10^3^ cells per well in 96-well plates. The corresponding interventions were added after the cells had adhered for 24 h. In each well, 10 μL of CCK-8 reagent was added, followed by a 2-h long incubation at 37 °C in the dark. The absorbance was measured at 450 nm with a microplate reader (Multiskan, FC, USA).

### 4.4. Western Blot Analysis

Western blot analysis was carried out as described previously by Wu et al. [25]. Briefly, proteins were separated using 10% SDS-PAGE gels and transferred onto PVDF membranes. After blocking with 5% skim milk, the membranes were incubated at 4 °C overnight with specific primary antibodies (both diluted 1:1000), followed by corresponding secondary antibodies. A chemiluminescence imaging system (Tanon 5200 Multi 4600SF, Shanghai, China) was employed for the visual analysis of protein bands. We quantified protein bands using Image J software (NIH, Bethesda, MD, USA), and the resulting data was input into GraphPad Prism 8.0 for visual analysis. The results were reported as mean ± standard deviation. Analysis of variance was used to assess mean differences between groups, and *p* values < 0.05 were considered statistically significant. All experiments were carried out separately in triplicate, and three technical replication samples from each independent experiment were Western blotted and quantified.

### 4.5. Reactive Oxygen Species Assay of Cells

The cells were given the appropriate interventions and exposed to 10 µmoL/L DCFH-DA (Beyotime Biotechnology Co., Ltd., S0033S, Shanghai, China) for 20 min. After three washes with serum-free cell culture media, the fluorescence intensity was quantified using flow cytometry (Beckman Coulter Cytoflex S, Brea, CA, USA).

### 4.6. Oil Red O Staining

The cells were washed three times with PBS and fixed with 4 percent paraformaldehyde for 30 min after the respective treatments. A concentration of 0.5% Oil Red O (Beijing Solarbio Science & Technology Co., Ltd., G1260, Beijing, China) was used to counterstain the cells for an hour before being imaged under an optical microscope (Nikon TS2, Tokyo, Japan). Image J analysis software was further utilized to examine the experimental data. We recorded the number of lipid droplets bound to Oil Red O in each group of cells. The ratio of each group to the control group was used as a multiple of the relative control group to analyze the differences between the groups.

### 4.7. Transmission Electron Microscopy (TEM)

The cells were collected and fixed for four hours in a glutaraldehyde solution containing 2.5 percent glutaraldehyde. Then, the cells were further fixed using 1 percent Osmium (VIII) oxide (OSO4). Dehydration of acetone on a scale of one to ten. A transmission electron microscope was used to observe and photograph the uranyl acetate and the cells stained with lead citrate (JEOL JEM-1400Flash, Tokyo, Japan).

### 4.8. Statistical Analysis

All experiments were conducted independently in triplicate, and the results were expressed as mean ± standard deviation. For statistical analysis, GraphPad Prism 8.0 was utilized. The mean difference between groups was determined using analysis of variance, and a *p*-value < 0.05 was considered statistically significant.

## 5. Conclusions

**Figure 6:**
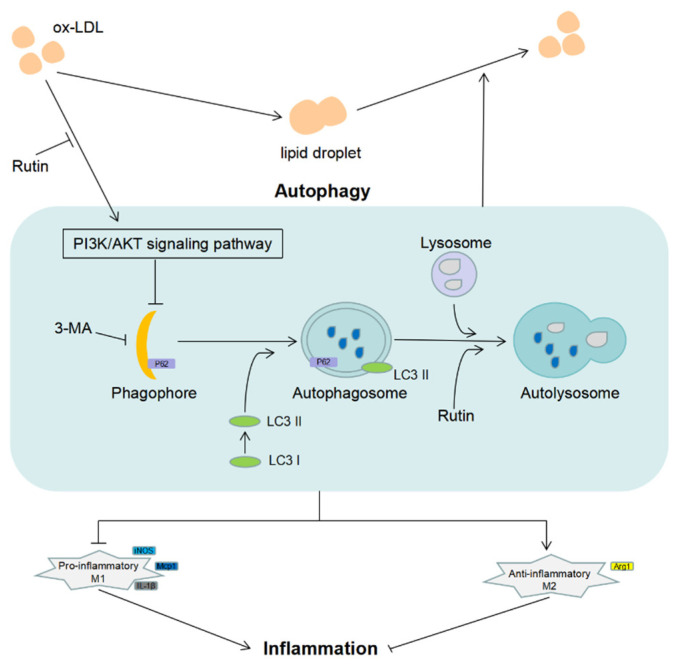
Schematic diagram of the anti-atherosclerosis effect of rutin.

Taken together, our findings indicate that rutin could effectively inhibit ox-LDL-mediated macrophage inflammation and foam cell generation, which are both associated with autophagy activation and regulation of the PI3K/ATK signaling pathway. Rutin might be potentially useful to treat atherosclerosis by decreasing macrophage inflammation and the generation of foam cells (Figure 6).

## Data Availability

The data used to support the findings of this study are available from the corresponding authors upon request.

## Abbreviations

AS	Atherosclerosis
CCK-8	Cell Counting Kit 8
DMEM	Dulbecco’s Modified Eagle’s Medium
FBS	Fetal Bovine Serum
OSO4	Osmium (VIII) oxide
ox-LDL	Oxygenated Low-density Lipoprotein
ROS	Reactive Oxygen Species
TEM	Transmission Electron Microscopy
3-MA	3-Methyladenine

## References

[1] Zhu Y., Xian X., Wang Z., Bi Y., Chen Q., Han X., Tang D., Chen R. (2018). Research Progress on the Relationship between Atherosclerosis and Inflammation. Biomolecules.

[2] Pedro-Botet J., Climent E., Benaiges D. (2020). Atherosclerosis and inflammation. New therapeutic approaches. Med. Clin..

[3] Maguire E.M., Pearce S.W.A., Xiao Q. (2019). Foam cell formation: A new target for fighting atherosclerosis and cardiovascular disease. Vascul. Pharmacol..

[4] Glick D., Barth S., Macleod K.F. (2010). Autophagy: Cellular and molecular mechanisms. J. Pathol..

[5] Zhou M., Ren P., Zhang Y., Li S., Li M., Li P., Shang J., Liu W., Liu H. (2019). Shen-Yuan-Dan Capsule Attenuates Atherosclerosis and Foam Cell Formation by Enhancing Autophagy and Inhibiting the PI3K/Akt/mTORC1 Signaling Pathway. Front. Pharmacol..

[6] Zhang X., Qin Y., Wan X., Liu H., Lv C., Ruan W., He L., Lu L., Guo X. (2021). Rosuvastatin exerts anti-atherosclerotic effects by improving macrophage-related foam cell formation and polarization conversion via mediating autophagic activities. J. Transl. Med..

[7] Miao J., Zang X., Cui X., Zhang J. (2020). Autophagy, Hyperlipidemia, and Atherosclerosis. Adv. Exp. Med. Biol..

[8] Ghorbani A. (2017). Mechanisms of antidiabetic effects of flavonoid rutin. Biomed. Pharmacother..

[9] Ma Y., Yang L., Ma J., Lu L., Wang X., Ren J., Yang J. (2017). Rutin attenuates doxorubicin-induced cardiotoxicity via regulating autophagy and apoptosis. Biochim. Biophys. Acta Mol. Basis. Dis..

[10] Li Y., Qin R., Yan H., Wang F., Huang S., Zhang Y., Zhong M., Zhang W., Wang Z. (2018). Inhibition of vascular smooth muscle cells premature senescence with rutin attenuates and stabilizes diabetic atherosclerosis. J. Nutr. Biochem..

[11] Xie Z., Wang X., Liu X., Du H., Sun C., Shao X., Tian J., Gu X., Wang H., Tian J. (2018). Adipose-Derived Exosomes Exert Proatherogenic Effects by Regulating Macrophage Foam Cell Formation and Polarization. J. Am. Heart Assoc..

[12] Geng J., Yang C., Wang B., Zhang X., Hu T., Gu Y., Li J. (2018). Trimethylamine N-oxide promotes atherosclerosis via CD36-dependent MAPK/JNK pathway. Biomed. Pharmacother..

[13] Lu S., Luo Y., Sun G., Sun X. (2020). Ginsenoside Compound K Attenuates Ox-LDL-Mediated Macrophage Inflammation and Foam Cell Formation via Autophagy Induction and Modulating NF-kappaB, p38, and JNK MAPK Signaling. Front. Pharmacol..

[14] Qiao L., Ma J., Zhang Z., Sui W., Zhai C., Xu D., Wang Z., Lu H., Zhang M., Zhang C. (2021). Deficient Chaperone-Mediated Autophagy Promotes Inflammation and Atherosclerosis. Circ. Res..

[15] Cao H., Jia Q., Yan L., Chen C., Xing S., Shen D. (2019). Quercetin Suppresses the Progression of Atherosclerosis by Regulating MST1-Mediated Autophagy in ox-LDL-Induced RAW264.7 Macrophage Foam Cells. Int. J. Mol. Sci..

[16] Fang S., Sun S., Cai H., Zou X., Wang S., Hao X., Wan X., Tian J., Li Z., He Z. (2021). IRGM/Irgm1 facilitates macrophage apoptosis through ROS generation and MAPK signal transduction: Irgm1 (+/−) mice display increases atherosclerotic plaque stability. Theranostics.

[17] Zhang M., Zhu R., Zhang L. (2020). Triclosan stimulates human vascular endothelial cell injury via repression of the PI3K/Akt/mTOR axis. Chemosphere.

[18] Wang Y., Lu Y.H., Tang C., Xue M., Li X.Y., Chang Y.P., Cheng Y., Li T., Yu X.C., Sun B. (2019). Calcium Dobesilate Restores Autophagy by Inhibiting the VEGF/PI3K/AKT/mTOR Signaling Pathway. Front. Pharmacol..

[19] Sun Y., Qin H., Zhang H., Feng X., Yang L., Hou D.X., Chen J. (2021). Fisetin inhibits inflammation and induces autophagy by mediating PI3K/AKT/mTOR signaling in LPS-induced RAW264.7 cells. Food Nutr. Res..

[20] Zhuo X., Wu Y., Yang Y., Gao L., Qiao X., Chen T. (2019). Knockdown of LSD1 meliorates Ox-LDL-stimulated NLRP3 activation and inflammation by promoting autophagy via SESN2-mesiated PI3K/Akt/mTOR signaling pathway. Life Sci..

[21] Libby P. (2021). The changing landscape of atherosclerosis. Nature.

[22] Varghese J.F., Patel R., Singh M., Yadav U.C.S. (2021). Fisetin Prevents Oxidized Low-density Lipoprotein-Induced Macrophage Foam Cell Formation. J. Cardiovasc. Pharmacol..

[23] Lin P., Ji H.H., Li Y.J., Guo S.D. (2021). Macrophage Plasticity and Atherosclerosis Therapy. Front. Mol. Biosci..

[24] Fei J., Sun Y., Duan Y., Xia J., Yu S., Ouyang P., Wang T., Zhang G. (2019). Low concentration of rutin treatment might alleviate the cardiotoxicity effect of pirarubicin on cardiomyocytes via activation of PI3K/AKT/mTOR signaling pathway. Biosci. Rep..

[25] Wu Q., Zhang B., Li B., Cao X., Chen X., Xue Q. (2020). PTBP3 promotes migration of non-small cell lung cancer through regulating E-cadherin in EMT signaling pathway. Cancer Cell Int..

